# Correlation between Trop2 and amphiregulin coexpression and overall survival in gastric cancer

**DOI:** 10.1002/cam4.1018

**Published:** 2017-03-03

**Authors:** Wei Zhao, Guipeng Ding, Jinbo Wen, Qi Tang, Hongmei Yong, Huijun Zhu, Shu Zhang, Zhenning Qiu, Zhenqing Feng, Jin Zhu

**Affiliations:** ^1^ Department of PathologyNanjing Medical UniversityNanjing210029China; ^2^ School of Public HealthNantong UniversityNantong226019China; ^3^ Department of EpidemiologySchool of Public HealthNanjing Medical UniversityNanjing210029China; ^4^ Key Laboratory of Antibody Technique of Ministry of HealthNanjing Medical UniversityNanjing210029China; ^5^ Department of OncologyHuai'an Hospital Affiliated of Xuzhou Medical College and Huai'an Second People's HospitalHuai'an223001China; ^6^ Department of PathologyAffiliated Hospital of Nantong UniversityNantong226019China; ^7^ Jiangsu Collaborative Innovation Center for Cancer Personalized MedicineNanjing Medical UniversityNanjing210029China; ^8^ Key Laboratory of Cancer BiomarkersPrevent and TreatmentCancer CenterNanjing Medical UniversityNanjing210029China; ^9^ Huadong Medical Institute of BiotechniquesNanjing210029China

**Keywords:** *AREG*, *coexpression*, *correlation*, *gastric cancer*, *Trop2*

## Abstract

Gastric cancer (GC) is a multistep and multistage disease and the majority of GC cells could overexpressed one or more oncogenes. Trop2 and amphiregulin (AREG) are both overexpressed in various epithelial cell cancers and have the role in the increases tumor cells division and metastasis. However, little is known about the function and correlation of two oncogenes coexpressed in GC. The expression level of these two genes in 791 cases of GC tissues were tested, the correlations between two genes expression and clinical pathological characteristics and overall survival in GC patients through immunohistochemistry (IHC) were analyzed. This study also explored the mRNA expression level of two genes in 26 cases of freshly GC tissues by qRT‐PCR. The results indicated that Trop2+/AREG+ coexpression was higher in GC tissues than in adjacent tissues. Trop2+/AREG+ protein coexpression were associated with Tumor Node Metastasis (TNM) stage (*χ* ^2^ = 50.345, *P *<* *0.001), tumor size (*χ* ^2^ = 40.349, *P *<* *0.001), lymph node metastases (*χ* ^2^ = 26.481, *P *<* *0.001), and distant metastases (*χ* ^2^ = 8.387, *P *=* *0.039). GC patients with Trop2+ and AREG+ protein coexpression had poor overall survival rates (HR = 3.682, 95% CI = 2.038–6.654, *P *<* *0.001). The expression level of Trop2/AREG were positively correlated (*r* 0.254 and *P *<* *0.001). The result of the mRNA expression was similar to that of the protein expression. Overall, Trop2 and AREG could be seen as prognostic cobiomarker in GC and combined detection of Trop2 and AREG could be viewed as helpful in predicting the prognosis of the GC patients.

## Introduction

Gastric cancer (GC) is one of the five common types of tumors and ranks the third leading cause of cancer‐related mortality worldwide 1. In Asian population, GC is the most common tumor and the majority of patients are diagnosed at an advanced stage 2, 3. The incidence of GC is also higher in China and GC ranks the second incidence in males and the fourth in females. Nowadays, due to the improvement of the health condition and eating habits, the incidence of GC has declined. But because of high rates of metastasis and recurrence, the 5‐years survival rate of the advanced stage patients is less than 20% 4. Thus, identifying novel molecular genetic markers to help diagnose GC earlier so as to improve clinical outcome of GC and understand the mechanism of tumorigenesis is very urgent.

The human trophoblast cell surface glycoprotein (TACSTD2/Trop2/M1S1/GA733‐1) was first identified in the surface of the human trophblast cells and located at chromosome 1q32 5. Trop2 is mainly expressed in the surface of the epithelial cells and is about 35‐kDa single pass transmembrane protein, which spans the cellular membrane: it has an extracellular, a transmembrane and an intracellular domain, along with a cytoplamic tail essential for signaling 5. Trop2 is overexpressed in various epithelial cell cancers 6, 7, 8. When cells become malignant, metastasis and recurrence, Trop2 is mainly expressed in the cytoplasm and could promote tumor proliferation 6, 9. On the contrary, somatic adult tissues show little or no Trop2 expression 6, 9, 10.

AREG is a member of epidermal growth factor (EGF) family and has a critical role in growth control, although the precise role has not been understood deeply. AREG is located at chromosome 4q13.3 and shedding ECD of original a 252 amino acid transmembrane precursor (pre‐AREG), then secret into blood or other cell surrounding microenvironment through autocrine and paracrine. Emerging evidence showed that AREG may be important for tumor metastasis and resistance to therapy 11, 12, 13. It has also been reported that overexpression of the AREG in cancer maybe related with resistance of conventional chemotherapeutic agents 14, 15, 16.

Our lab have been studied two papers about the expression of two genes in gastric cancer, respectively 17 (Bing W,2016,oncotarget, accepted) and found Trop2 and AREG was all overexpression in GC tissues. But because of the complexity of the cancer microenvironment in the solid tumor 18, the majority of the cancer cells could overexpress two or more oncogenes in the meantime 19. This is also bring a difficult problem toward the tumor target therapy in solid tumors. So this study was to investigate the expression of these two oncogenes in GC tissues and examine the relationship with clinicalpathological characteristics as well as overall survival in GC patients.

## Methods

### Human tissue specimens and patient clinical information

A total of 791 formalin‐fixed, paraffin‐embedded (FFPE) stomach tissue samples were collected from 741 patients, including 589 cancer, 89 matched tumor neighbor, 65 chronic gastritis, 26 intestinal metaplasia, and 22 intraepithelial neoplasia. All FFPE specimens were obtained from the Department of Pathology, Affiliated Hospital of Nantong University from 2003 to 2010. The associated clinical records of the donor patients including age, sex, tumor node metastasis (TNM) stage, histological type, differentiation status. No patients received any types of treatments (radiation therapy, chemotherapy, or immunotherapy) before surgery. Overall survival (OS) was defined as the period from initial biopsy confined diagnosis to death. Information on patients who were alive at the last follow‐up date was deleted from the analysis and follow‐up from 2 to 10 years. Additional 26 freshly gastric cancer and matched tumor neighbor tissues were obtained primarily from the First Affiliated Hospital of Nanjing Medical University, Huai'an Second People's Hospital, and Affiliated Hospital of Nantong University. The study protocol was approved by the Human Research Ethics Committees of these hospitals. All patients provided written informed consent for their stomach tissue samples to be used for research.

### Quantitative real‐time polymerase chain reaction (qRT‐PCR)

Total RNA was extracted from freshly samples using TRIzol reagent (Invitrogen, Carlsbad, CA) and reverse transcribed into cDNA using a PrimeScript^™^ RT reagent kit (Takara, Glen Burnie, MD) in accordance with the manufacturer's instructions. Human *β*‐actin served as the internal control for determining Trop2 and AREG mRNA levels. The primers as follows: human *β*‐actin forward, 5′‐ TGGAGAAAATCTGGCACCAC‐3′, and reverse, 5′‐GATGATGCCTCGTTCTAC‐3′, and Trop2 forward, 5′‐TGTCCTGATGTGATATGTCTGAG‐3′, and reverse, 5′‐GGGTGAGAGTGGGTTGGG‐3′, AREG primer: forward, 5′‐GCTGTCGCTCTTGATACTCG‐3′, and reverse,5′‐.

ACGCTTCCCAGAGTAGGTGT‐3′ (Genescript. Nanjing, China). Comparative quantification was determined using the 2^−∆∆Ct^ Method.

### Statistical analysis

The data for statistical analyses were performed by using the SPSS 18.0 statistical software package (SPSS Inc., Chicago, IL). The Student's t‐test and Pearson *χ* ^2^ test were performed to determine the statistical significance of differences groups. The X‐tile software program (The Rimm Lab at Yale University; http://www.tissuearray.org/rimmlab) 20 was conducted for statistical analysis of immunohistochemistry (IHC) data after converting it into dichotic data (“low or no” *vs*. “high”) using predeterminate cut‐off values 21. Both Kaplan–Meier method and a log‐rank test were implemented to evaluate the significant difference of overall survival of patients. The univariate and multivariate hazard ratios for the variables were analyzed by a cox proportional hazards model. The correlation analysis between Trop2 and AREG was carried out by Spearman correlation test. A two‐tailed *P*‐value of less than 0.05 was considered as statistically significant.

## Results

### Trop2 and AREG expression in GC tissues

**Figure 1:**
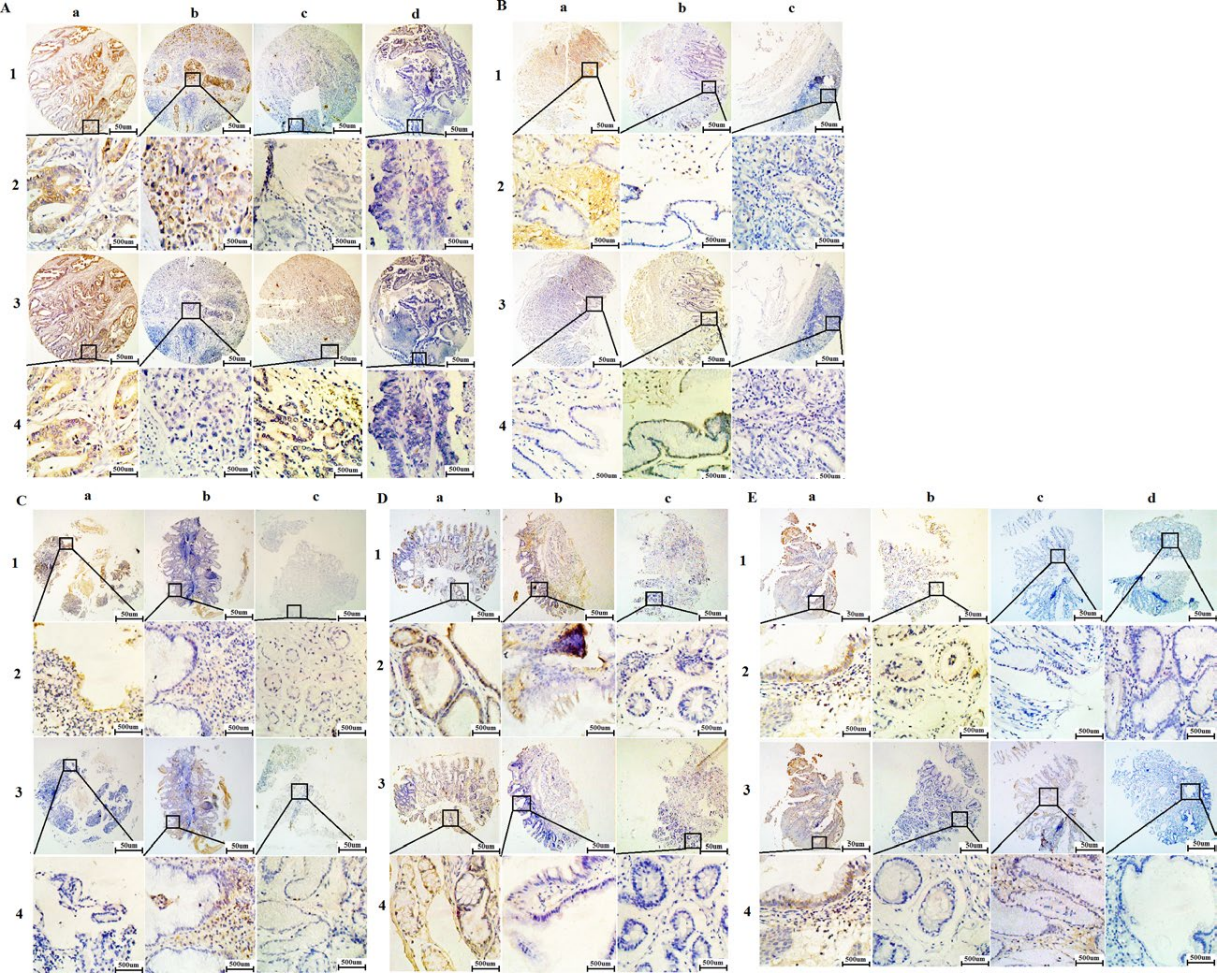
Representative images of Trop2 and AREG protein expression in gastric tissues TMA sections. (A)Gastric cancer with expression of Trop2 and AREG: (a1‐2) high expression of Trop2 immunohistochemistry (IHC score, 300); (a3‐4) high expression of AREG (IHC score, 80); (b1‐2) high expression of Trop2(IHC score, 200); (b3‐4)low expression of AREG (IHC score, 10);(c1‐2) low expression of Trop2(IHC score, 30) (c3‐4) high expression of AREG (IHC score, 150); (d1‐2) low expression of Trop2 (IHC score, 50) (d3‐4) low expression of AREG (IHC score, 50). (B) Matched adjacent tissue with expression of Trop2 and AREG: (a1‐2) high expression of Trop2 (IHC score, 200); (a3‐4) low expression of AREG (IHC score,50) (b1‐2) low expression of Trop2(IHC score, 60); (b3‐4)high expression of AREG (IHC score, 150);(c1‐2) low expression of Trop2(IHC score, 60) (c3‐4) low expression of AREG (IHC score, 0). (C) Chronic gastritis with expression of Trop2 and AREG: (a1‐2) high expression of Trop2 (IHC score, 150); (a3‐4) low expression of AREG (IHC score, 0); (b1‐2) low expression of Trop2 (IHC score, 100); (b3‐4) high expression of AREG (IHC score, 160); (c1‐2) low expression of Trop2 (IHC score, 30); (c3‐4)low expression of AREG (IHC score, 20). (D) Intestinal metaplasia with expression of Trop2 and AREG: (a1‐2) high expression of Trop2 (IHC score, 180); (a3‐4) high expression of AREG (IHC score, 200); (b1‐2) high expression of Trop2 (IHC score, 150); (b3‐4) low expression of AREG (IHC score, 10); (c1‐2) low expression of Trop2 (IHC score, 30); (c3‐4) low expression of AREG (IHC score, 10). (E) Intraepithelial neoplasia with expression of Trop2 and AREG: (a1‐2) high expression of Trop2 (IHC score, 200); (a3‐4) high expression of AREG (IHC score, 160); (b1‐2) high expression of Trop2 (IHC score, 200); (b3‐4) low expression of AREG(B3‐4) (IHC score, 10);(c1‐2) low expression of Trop2 (IHC score, 0); (c3‐4) high expression of AREG (IHC score, 130); (d1‐2) low expression of Trop2 (IHC score,10); (d3‐4) low expression of AREG (D3‐4) (IHC score, 0).

Trop2 and AREG, which were located at the membrane and cytoplasm through IHC, were all overexpressed in GC tissues. Using X‐tile software program for TMA data analysis (http://www.tissuearray.org/rimmlab), we identified the cut‐off point according to overall survival in GC tissues. For Trop2 and AREG, the cutoff 130 was selected:0–130 was considered “low or no” expression, whereas 131–300 was considered “high” expression. High Trop2 and high AREG (T+A+), high Trop2 and low AREG (T+A‐), low Trop2 and high AREG (T‐A+), low Trop2 and low AREG (T‐A‐) expression were detected, respectively, in gastric tissues, which including cancer, matched adjacent tissue, chronic gastritis, intestinal metaplasia, intraepithelial neoplasia (Fig. 1). The level of T+A+, T+A‐, T‐A+, and T‐A‐ expression in cancer tissues were 41.42%, 24.62%, 21.39%, and 12.56%, respectively. T‐A‐express level were found the minimum than other levels in cancer tissues (Table 1). Interestingly, T+A+ expression did not detect in adjacent tissues and chronic gastritis tissues.

**Table 1:** Trop2 and AREG expression in gastric tissues.+: represents high expression, ‐: represents Low or no expression

Characteristic	*n*	Trop2	+	+	−	−	*χ* ^2^	*P*
		AREG	+	−	+	−		
Stomach							222.027	<0.001*
Cancer	589		244 (41.42)	145 (24.62)	126 (21.39)	74 (12.56)		
Matched adjacent tissue	89		0 (0.00)	29 (32.58)	28 (31.46)	32 (35.96)		
Chronic gastritis	65		0 (0.00)	8 (12.31)	7 (10.77)	50 (76.92)		
Intestinal metaplasia	26		11 (42.31)	12 (46.15)	0 (0.00)	3 (11.44)		
Intraepithelial neoplasia	22		3 (13.64)	5 (22.73)	6 (27.27)	8 (36.36)		

*χ*2 and *P* values for stomach overall includes all types gastric tissues. *P < 0.05.

### Association of Trop2 and AREG expression with clinicopathologic characteristics in GC

This study was also collected the data of GC patients’ clinicopathologic characteristics and examined the relationship between Trop2 and AREG protein expression and clinicopathologic parameter in GC patients (Table 2).The result indicated that T+A+ expression in GC was significantly associated with TNM stage (*χ* ^2^ = 50.345, *P *<* *0.001), tumor size(*χ* ^2^ = 40.349, *P *<* *0.001), lymph node metastases (*χ* ^2^ = 26.481, *P *<* *0.001), and distant metastases(*χ* ^2^ = 8.387, *P *=* *0.039). However, no correlation was found between Trop2 and AREG expression levels and gender, age, histological type, differentiation (Table 2).

**Table 2:** Association of high expression of Trop2 and AREG with clinicopathologic characteristics in GC patients

Characteristic	*n*	Trop2	+	+	−	−	Pearson *χ* ^2^	*P*
		AREG	+	−	+	−		
Total	589							
Gender							1.872	0.599
Male	421		177 (42.04)	99 (23.52)	95 (22.57)	50 (11.88)		
Female	168		67 (39.89)	46 (27.38)	32 (19.05)	23 (13.69)		
Age							5.418	0.144
<60	325		134 (41.23)	90 (27.69)	61 (18.77)	40 (12.31)		
≥60	264		110 (41.67)	55 (20.83)	66 (25.00)	33 (12.50)		
Histological type							15.143	0.234
Tubular	516		215 (41.67)	125 (24.22)	115 (22.29)	61 (15.70)		
Mixed (tubular and mucinous)	7		1 (14.29)	1 (14.29)	4 (57.14)	1 (14.29)		
Mucinous	32		15 (46.88)	8 (25.00)	5 (15.63)	4 (12.50)		
Signet ring cell	22		6 (27.27)	4 (18.18)	6 (27.27)	6 (27.27)		
Others^a^	12		7 (58.33)	4 (33.33)	0 (0.00)	1 (8.33)		
Differentiation							13.805	0.129
Well	57		18 (31.58)	10 (17.54)	19 (33.33)	10 (17.54)		
Moderate	141		59 (41.84)	30 (21.28)	36 (25.53)	16 (11.35)		
Poor	325		142 (22.72)	87 (26.77)	60 (18.46)	36 (11.08)		
Others^b^	66		25 (37.88)	18 (27.27)	12 (18.18)	11 (16.67)		
TNM stage							50.345	<0.001^c^
0	18		6 (33.33)	2 (11.11)	8 (44.44)	2 (11.11)		
Ia + Ib	110		33 (30.00)	16 (14.55)	39 (35.46)	22 (20.00)		
IIa + IIb	205		79 (38.54)	55 (26.83)	48 (23.41)	23 (11.22)		
IIIa+ IIIb	203		95 (46.80)	61 (30.05)	24 (11.82)	23 (11.33)		
IIIc + IV	53		31 (58.49)	11 (20.75)	8 (15.09)	3 (5.66)		
Tumor size							40.349	<0.001^c^
T0	18		6 (33.33)	2 (11.11)	8 (44.44)	2 (11.11)		
T1a+T1b+T2	179		53 (29.61)	36 (20.11)	58 (32.40)	32 (17.88)		
T3+T4a+T4b	392		185 (47.19)	107 (27.30)	61 (15.56)	39 (9.95)		
Lymph node metastases							26.481	<0.001^c^
N0	224		84 (37.50)	39 (17.41)	71 (31.70)	30 (13.39)		
N1	365		160 (43.84)	106 (29.04)	56 (15.34)	43 (11.78)		
Distant metastases							8.387	0.039
M0	551		222 (40.29)	134 (24.32)	123 (22.32)	72 (13.07)		
M1	38		22 (57.89)	11 (28.95)	4 (10.53)	1 (2.63)		

TNM, Tumor Node Metastasis.

a others include: pallipary adenocarcinoma, 3 cases; adeno‐squamous carcinoma, 3cases; squamous cell carcinoma, 3 cases; undifferentiated carcinoma, 2 cases; and neuroendocrine carcinoma, 1 case.

b others include everything besides tubular and papillary adenocarcinoma.

c *P < *0.05.

### The association between overexpression Trop2/AREG protein and the prognosis in GC

**Figure 2:**
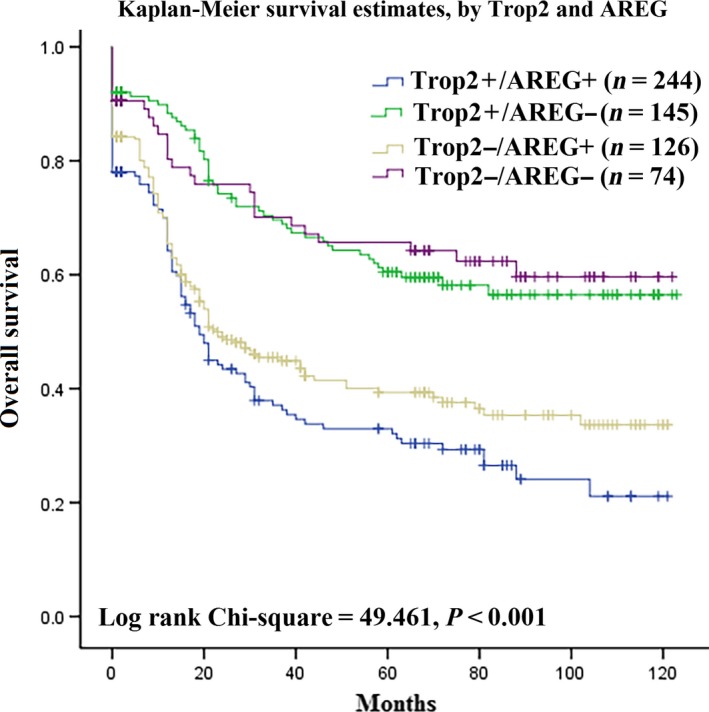
Survival curves for gastric cancer using the Kaplan–Meier method and the log‐rank test. Overall survival curves for patients with Trop2 + AREG+ expression (blue line,1), Trop2 + AREG‐(green line, 2), Trop2‐AREG+(gray line, 3) and Trop2‐AREG‐(purple line, 4).

Furthermore, the results of univariate and multivariate analyses indicated that Trop2 and AREG protein expression was correlated with prognostic factors in GC (Table 3). The result showed that high level of Trop2/AREG expression and TNM stage were associated with poor survival with HR = 3.682, 95% CI = 2.038–6.654, *P *<* *0.001and HR = 6.387, 95% CI = 2.868–14.221, *P *<* *0.001, respectively, in univariate analysis. The similar result was also found in multivariate analysis. The results of Kaplan–Meier survival curves showed that the group with high level of Trop2 and AREG expression was significantly associated with poor overall survival. In contrast, no statistically significant interactions were observed between group with solo high level of Trop2 protein expression and poor overall survival (Fig. 2). These results showed that high level of Trop2 and AREG expression could be more suitable as prognostic factor than solo high level of Trop2 or solo AREG expression group for GC progress.

**Table 3:** Univariate and multivariate analysis of prognostic markers for overall survival in gastric cancer

	Univariate analysis			Multivariate analysis		
	HR	*P *> |z|	95% CI	HR	*P *> |z|	95% CI
Trop2 and AREG						
T + A + versus T + A‐versus T‐E + versus T‐A‐	3.682	<0.001^b^	2.038–6.654	3.104	0.001^b^	1.590–6.061
Age						
<60 versus ≥60	1.303	0.178	0.886–1.916	–	–	–
Gender						
Male versus Female	0.939	0.730	0.654–1.347	–	–	–
Histological type						
Tubular versus Mixed(tubular and mucinous) versus Mucinous versus signet ring cells versus others^a^	0.083	0.119	0.011–1.323	–	–	–
Differentiation						
Well versus Moderate versus Poor	1.722	0.137	0.842–3.524	–	–	–
TNM stage						
0 versus Ia + Ib versus IIa + IIb versus IIIa + IIIb versus IIIc + IV	6.387	<0.001^b^	2.868–14.221	6.848	<0.001^b^	3.028–15.486
Tumor size						
Tis versus T1 versus T2 versus T3 versus T4	4.181	<0.001^b^	2.873–6.085	–	–	–
Lymph node metastases						
N0 versus N1 versus N2 versus N3	4.761	<0.001^b^	3.330–6.806	–	–	–
Distant metastases						
M0 versus M1	7.323	<0.001^b^	2.564–20.914	–	–	–

TNM, Tumor Node Metastasis

a others include: pallipary adenocarcinoma, 3 cases; adeno‐squamous carcinoma, 3 cases; squamous cell carcinoma, 3 cases; undifferentiated carcinoma, 2 cases; and neuroendocrine carcinoma, 1 case.

b *P *<* *0.05.

### The correlation analysis of the expression level between Trop2 and AREG in GC

**Figure 3:**
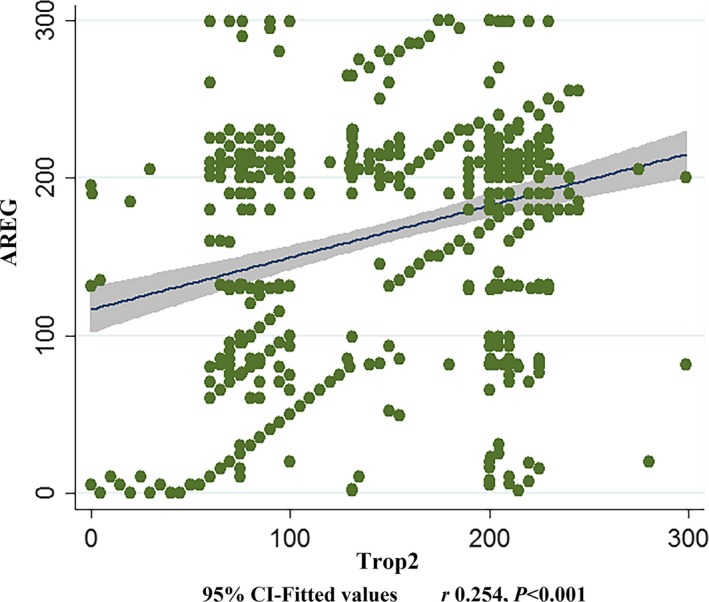
Scatter diagram for correlation analysis of the expression level between Trop2 and AREG in GC

Spearman correlation analysis was used to analysis the correlation between TMA data of Trop2 and AREG, which were continuous variables without normal distribution. From Figure 3, it was indicated that these two continuous variables show significant positive correlation with *r* 0.254 and *P *<* *0.001.

### The correlation analysis of the mRNA expression level between Trop2 and AREG in GC tissues

**Figure 4:**
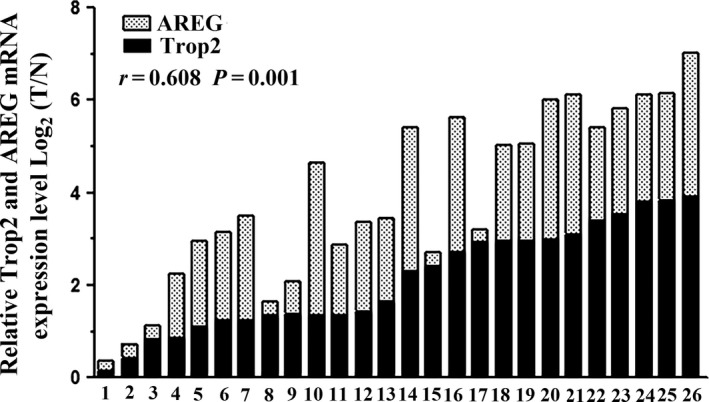
Trop2 and AREG mRNA expression in 26 pairs GC tissue pairs. Trop2 and AREG mRNA expression was examined by qRT‐PCR and normalized to *β*‐actin. T=GC tissues; N=matched tumor neighbor tissues.

The qRT‐PCR was performed in 26 pairs of fresh GC tissues and matched tumor neighbor tissue to further investigated the correlation between Trop2 and AREG expression in GC. Trop2 and AREG mRNA level were 2.12 ± 1.14, 1.80 ± 1.03 fold higher in GC tissues than in matched tumor neighbor tissues, respectively (*P *<* *0.001, Fig. 4). Meanwhile, these two continuous variables showed significant positive correlation with *r* 0.608 and *P *=* *0.001, the result of the mRNA expression was similar to that of the protein expression.

## Discussion

This study retrospectively evaluated the coexpression levels of Trop2 and AREG on the gastric tissues. The results revealed an association between Trop2 and AREG over expression and poor prognostic in GC tissues by IHC analysis on tissue microarray (TMA). Both Trop2+ and AREG+ group coexpression level was the highest (41.42%) than T+A‐(24.62%), T‐A+(21.39%) and T‐A‐(12.56%) groups in cancer tissues and the T+A+ expression level in cancer tissues were higher than that in the matched adjacent tissue. The results also indicated that high Trop2 and AREG protein coexpression was associated with TNM stage, lymph node metastases and distant metastases. Furthermore, high level of Trop2 and AREG expression (T+A+) predicted poor overall survival. The expression level of two genes was positively correlative in GC tissues, and the similar results were found with regard to the mRNA expression level between two genes.

It is well‐known that GC was developed from normal gastric mucosa to chronic gastritis, chronic atronic gastritis, intestinal metaplasia, atypical hyperplasia (low‐grade intraepithelial neoplasia, and high‐grade intraepithelial neoplasia) to cancer. The turning point of malignancy changes is the status of intestinal metaplasia 22.

The overexpression of oncogene Trop2 in several types of solid tumor cancers had been provided 23, 24, 25, such as oral squamous epithelial cell carcinoma 26, breast cancer 27, and cervical cancer 24. It is also upregulated in certain malignant hematological diseases, including non‐Hodgkin's lymphoma (NHL) and leukemia. The effects of Trop2 need to be binding several parters: such as IGF‐1, claudin‐1 and 7, cyclin D1, and PKC. By these targets, Trop2 could maintaining tight junction integrity 5; increases tumor proliferation through activated the NF‐kB, cyclin D1 and ERK[5, 6, 7, 8, 28]; and suppresses IGF‐1R signaling 7.

AREG, as a ligand for the EGFR, is a transmemebrane tyrosine kinase receptor, which plays a central role in regulating cell division and death 29. Overexpression of AREG was found in several cancers, such as ovarian cancer, breast cancer, hepatocellular cancer, and gastric cancer 27. Whereas Ichikawa et al. 30reported the adverse result about AREG expression in gastric cancer through mRNA level that AREG gene expression was associated with good outcomes. But differ to our study, in their study the expression of 63 genes(including three reference genes) were examined in 829 samples of the gastric cancer tissues in GC patients from Japan, whom with stage II/III gastric cancer enrolled in the Adjuvant Chemotherapy Trial of S‐1 for Gastric. Because those GC patients were different from our collected the gastic tissue samples, which were did not received any treatment and the discrepancy need to be further studies.

This study has several limitations: first, this study is a retrospective observation examine and the gastric tissues of patients were obtained from 2003 to 2010, the conclusion might not be suitable for the current population; second, IHC used in this study is a semiquantitative method, additional study in vivo and in vitro should be done in the future to confirm the role of Trop2 and AREG in cancer cells; Third, the number of genes coexamined in this study were relatively small and other useful candidate genes should be studied.

In conclusion, high Trop2 and AREG expression is associated with poor prognosis and may be considered as an independent prognostic cobiomarker in GC.

## Conflict of Interest

The authors have declared that no competing financial interests exist.
